# Brief CommunicationCirculating tumor DNA is present in the most aggressive meningiomas

**DOI:** 10.1093/noajnl/vdaa068

**Published:** 2020-06-07

**Authors:** Thomas Graillon, Catherine Roche, Noémie Basset, Gregory Mougel, Mikael Meyer, Kaissar Farah, Sébastien Boissonneau, Stéphane Fuentes, Emeline Tabouret, Chantal Campello, Romain Appay, Dominique Figarella-Branger, Olivier Chinot, Henry Dufour, Pauline Romanet, Anne Barlier

**Affiliations:** 1 Department of Neurosurgery, Hospital La Timone, Aix Marseille Univ, APHM, INSERM, MMG, Marseille, France; 2 Laboratory of Molecular Biology, Hospital La Conception, Aix Marseille Univ, APHM, INSERM, MMG, Marseille, France; 3 Department of Neurosurgery, Hospital La Timone, Aix Marseille Univ, APHM, Marseille, France; 4 Aix-Marseille Univ, APHM, CNRS, INP, Inst Neurophysiopathol, CHU Timone, Service de Neuro-chirurgie, Marseille, France; 5 Aix-Marseille Univ, APHM, CNRS, INP, Inst Neurophysiopathol, CHU Timone, Service de Neuro-Oncologie, Marseille, France; 6 Aix-Marseille Univ, APHM, CNRS, INP, Inst Neurophysiopathol, CHU Timone, Service d’Anatomie Pathologique et de Neuropathologie, Marseille, France

Recent discoveries of multiple driver mutations open promising perspectives for targeted therapies in meningioma. Nevertheless, iterative recurrences of most aggressive meningiomas as extended skull base meningiomas are not systematically operated and histologically documented. This suggests the interest and the relevance of liquid biopsy in meningiomas. In a proof-of-concept study, we detected the *NF2* mutation in 2 of 6 cell-free plasma DNAs and 1 of 1 cerebrospinal fluid (CSF) from high-grade recurrent cases, suggesting that identification of the driver mutation in blood and CSF is today feasible. Liquid biopsy could be an interesting tool to adapt the targeted therapy in meningiomas in the near future.

Since the discovery of *NF2*’s involvement, many advances have been performed in the description of meningioma mutational landscape.^1^ The therapeutic management of refractory meningioma to iterative surgeries and radiotherapy sessions remains an unmet medical need in neurooncology. Targeted therapies were suggested to be of interest in aggressive meningiomas, particularly for skull base ones given the large pattern of newly discovered driver mutations, which could be targeted by specific inhibitors. Recently, a case of *AKT1*-mutated skull base meningioma treated by AKT1 inhibitor was reported to be successful.^2^ PI3K–Akt–mTOR pathway targeting was also demonstrated to be relevant in meningiomas.^3^

Although tumor tissue is likely available in recurrent high-grade meningiomas, iterative tumor recurrences are not systematically operated and then often non-histologically documented. Recent studies demonstrated that the mutational pattern of meningioma recurrence could differ from the initial tumor to remote recurrence.^1^ Moreover, complex skull base meningiomas are often nonaccessible to surgery, treated without histology, and yet could benefit from targeted therapies. Therefore, in these different situations, liquid biopsy could be of interest in the patient diagnosis, prognosis, and to guide therapeutic management.

The detection of tumor mutation in cell-free plasma DNA (cfDNA) constitutes the most promising biomarker in cancer. In meningioma, in contrast to other brain tumors, the lack of brain–blood barrier makes possible the passage of circulating tumor DNA (ctDNA) in the blood. As an alternative solution tumor mutations could be searched in the CSF. In a proof-of-concept study, we analyzed cfDNA in blood from 15 patients and in CSF from 3 other patients, all previously identified with at least one pathogenic variant in their tumor, except one (M17, Table 1).

**Table 1.** Clinical and Molecular Characteristics of Meningiomas

Tumor	Age (year) / Sex	Histological subtype	WHO grade	Tumoral location	Tumoral volume (cm^3^)	Tumoral extension	Number of surgical removal	Mutated gene^a^	Driver mutation(s) identified in tissue	Mutated allelic fraction^b^ in tissue (%)	Sample cfDNA/ CSF	Coverage of depth at the position of the driver mutation	Allelic fraction positive reads/total reads (%) in cfDNA or CSF^c^
Benign meningiomas													
M1	87/F	Meningothelial	I	Tuberculum	7	—	1	*TRAF7*	c.1606 G>A, p.(Gly536Ser)	36.7%	cfDNA	No	1759×
M2	58/F	Meningothelial	I	Frontal	16	—	1	*KLF4* *TRAF7*	c.1225A>C, p.(Gly559Cys) c.1657G>T, p.(Lys409Gln)	46.9% 41.8%	cfDNA	No No	2798× 2106×
M3	73/F	Atypical	II	Parasagittal	79	—	1	*NF2*	c.855dupT, p.(Asn286fs)	82.5%	cfDNA	No	1718×
M4	71/M	Atypical	II	Conv “en plaques”	18	—	1	*NF2*	c.734delA, p.(Asp245fs)	47.1%	cfDNA	No	2400×
M5	33/F	Transitional	I	Ant Skull base, multiple	57	—	1	*AKT1* *TRAF7*	c.49G>A, p.(Glu17Lys) c.1911G>C, p.(Gln637His)	31.7% 26.4%	cfDNA	No No	1296× 2600×
M6	85/F	Atypical	II	Temporal	10	—	1	*NF2*	c.448-1G>A, p.(?)	59.6%	cfDNA	No	1630×
M7	55/F	Meningothelial	I	Ant Skull base	25	—	1	*SMO*	c.1234C>T, p.(Leu412Phe)	28.5%	cfDNA	No	3941×
M8	68/F	Atypical	II	Parietal, multiple	56	—	1	*AKT1*	c.49G>A, p.(Glu17Lys)	35%	cfDNA	No	313×
M9	79/F	Meningothelial	I	Temporal	65	—	1	*NF2*	c.141delT, p.(Phe47fs)	47.3%	cfDNA	No	568×
M10	39/F	Psammous	I	Thoracic	3	—	2	*NF2*	c.737delC, p.(Pro246fs)	52.5%	CSF	No	1412×
M11	69/F	Atypical	II	Petrous bone	24	—	2	*NF2*	c.532C>T, p.(Glu178*)	36%	CSF	No	1072×
Recurrent high-grade meningiomas													
M12	50/M	Anaplastic	III	Occipital	71	Sub cut	2	*NF2*	c.134_135delAC, p.(Asp45fs)	41.8%	cfDNA	No	2807×
M13	53/M	Anaplastic	III	Temporal	148	—	3	*NF2*	c.1445delC, p.(Pro482fs)	58%	cfDNA	No	1129×
M14	80/F	Anaplastic	III	Fronto orb	15	Sub cut	2	*NF2*	c.819delT, p.(Lys274fs)	76%	cfDNA	Yes (1.4%)	550×
M15	75/F	Anaplastic	III	Intravent	29	LM	2	*NF2*	c.447 + 1G>A p.(?)	27%	cfDNA	No	1595×
M16	72/F	Anaplastic	III	Conv	190	Sub cut	6	*NF2*	c.110dupGA, p.(Cys37fs)	8%	cfDNA	Yes (3.7%)	4555×
M17	68/F	Atypical	II	CS, Parasellar	84	—	1	*NF2*	c.1341-2A>C, p.(?)	Not detected^d^	CSF	Yes (28.7%)	2890×
M18	66/M	Anaplastic	III	Parieto-occipital	6	—	2	*NF2*	c.432C>G, p.(Tyr144*)	64%	cfDNA	No	3129×

F, female; M, male, CS, cavernous sinus; conv, convexity; Sub cut, subcutaneous; LM, leptomeningeal; cfDNA, cell-free plasma DNA; CSF, cerebrospinal fluid; NGS, next-generation sequencing.

^a^The coding exons and exon–intron boundaries of 13 genes (*NF2* (NM_000268.3), *AKT1*(NM_001014431.1), *SMO* (NM_005631.4), *KLF4*(NM_004235.4), *TRAF7*(NM_032271.2), *PIK3CA*, *SUFU*, *SMARCB1* (NM_003073.3), *SMARCE1* (NM_003079.4), *CDKN2A* (NM_058195.3), *CDKN2B* (NM_004936.3), *PTEN* (NM_000314.4), and *TERT* (NM_198253.2) were sequenced using the Custom QIAseq targeted DNA Panel (Qiagen) on a MiSeqDx (Illumina) as previously described.

^b^The mutated allelic fraction is the count of mutated alleles out of the total number of alleles (wild type + mutated).

^c^Coverage of depth: number of times the nucleotide seat of the mutation in the tissue has been read by sequencing.

^d^No mutation detected in the tumor biopsy.

Eleven patients presented with benign meningioma (WHO grade I and II) and 7 with recurrent high-grade meningiomas were included. Written informed consent was required for each patient. The study was approved by the Aix-Marseille University IRB.

In all cases, the blood sample was taken before surgery. The CSF has been collected during surgery for thoracic benign meningioma (M10) and during ventriculoperitoneal shunt implantation in 2 cases (M11, benign and M17, aggressive). cfDNAs were extracted from 4 mL of serum and from 1.5 to 4 mL of CSF, all previously identified with at least one pathogenic variant in their tumor, except one (M17, Table 1).

Specimens were analyzed by ultradeep sequencing on a MiSeqDx (Illumina) using the Custom QIAseq targeted DNA Panel library preparation (Qiagen) targeting *NF2*, *AKT1*, *SMO*, *KLF4*, *TRAF7*, *PIK3CA*, *SUFU*, *SMARCB1*, *SMARCE1*, *CDKN2A*, *CDKN2B*, *PTEN*, and *TERT.* This library preparation is suitable for rare event detection because random molecular barcodes were incorporated to reduce the error rate and reach a sensitivity of mutation detection less than 0.1%.^4^ The bioinformatics analysis was carried out using smCounter2.^5^ The previously identified variants in tumor tissue were searched in the VCF files and in BAM files. In BAM files, we eliminated the background noise by seeing if the variant was present in the other samples. If it is present, we considered this variant as true only if its coverage of depth was more than the mean + 2 standard deviations of the other samples.

Among the 11 benign cases (9 cfDNAs and 2 CSF samples), we did not detect any of the previously identified mutations in the tumor (Table 1). Among the 7 aggressive cases (6 cfDNAs and 1 CSF sample), except patient M17, all the patients had a tumor biopsy with an *NF2* mutation. In 2 of 6 cases (M16 and M18), the *NF2* mutation was detected in cfDNA. In these 2 cases, a subcutaneous extension was observed as shown on MRI (Figure 1A and B). Moreover, an *NF2* mutation at a high allelic frequency (28.7%) was detected in the CSF from a patient bearing a very aggressive tumor (M17). This *NF2* mutation was not present in a tumor biopsy (cfDNA was not available). Interestingly, the patient underwent radiation therapy for cavernous sinus meningioma without tumor biopsy in 1999. She was referred for trigeminal neuralgia impairment in 2016. The MRI displayed slight tumor growth (Figure 1C and D). Re-irradiation was contra-indicated. One year later, she was referred for cognitive troubles and severe visual loss. The MRI displayed a dramatic tumor progression (Figure 1E–H). An endoscopic transsphenoidal biopsy and internal ventriculoperitoneal shunt implantation with CSF analysis were performed. She deceased many weeks later. Neuropathological conclusions were an atypical meningioma with meningothelial and secretory features.

**Figure 1.**
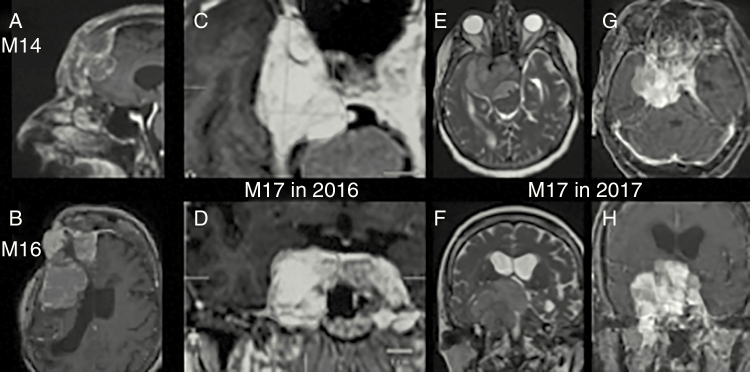
Patients with positive liquid biopsy. (A and B) Patients M14 (A) and M16 (B): 3D T1-weighted with gadolinium enhancement MRI. In both cases, the *NF2* mutation was detected in cfDNA. A subcutaneous extension was observed on MRI. (C–H) Patient M17: C and D display axial and coronal 3D T1-weighted with gadolinium enhancement MRI in 2016; E and F display axial and coronal T2-weighted MRI in 2017; G and H represent axial and coronal 3D T1-weighted with gadolinium enhancement MRI in 2017. We observed a major progression comparing tumor volume in 2016 and 2017. *NF2* mutation at a high allelic frequency (28.7%) was detected in the CSF.

It was the first study identifying ctDNA mutation driver from meningioma patients. A previous study analyzed cfDNA from 34 meningiomas.^6^ In this study, several variants have been identified, but no gene was a driver for meningioma. Moreover, no correlation was made with the tumor; therefore, the tumor origin of the identified variants cannot be asserted.^7^

In the patient M17, even with a good quality of biopsy material, no *NF2* genetic alteration was detected but the issue could be the area of tumor biopsy in this huge tumor. The lack of *NF2* mutation is frequent in skull base meningiomas with meningothelial and secretory features. Two hypotheses may be done to explain the *NF2* mutation detected in CSF: (1) a second genetic occurrence with an *NF2* mutation leading to extremely aggressive meningioma or (2) a second meningioma occurrence. However, the second hypothesis is not in accordance with the MRI showing a clear tumor progression (Figure 1).

In benign meningiomas, mutation detection in circulating DNA remains negative suggesting a low level of ctDNA in blood and CSF. In the aggressive tumors, mutation drivers were detectable in cfDNA from 2 of 6 patients and in CSF from 1 of 1 patient. Recurrent high-grade meningiomas represent a low part of meningiomas, but their therapeutic management remains particularly challenging today. The role of the subcutaneous invasion in ctDNA detection remains unknown and requires further studies. It has been shown that patients with tumors limited to the central nervous system have significantly enriched ctDNA in CSF.^8^ Interestingly, we were able to detect in CSF an *NF2* mutation not found in the tumor, a feature already reported in brain tumor,^8^ which may be due to the tumor heterogeneity.^9^ Further studies are required to assess the sensitivity and the interest of cfDNA analysis to address some limitations of tissue-based genetics.^10^

Circulating tumor DNA could be currently detected in the blood and the CSF of recurrent high-grade meningiomas. The presence of ctDNA in blood or CSF from meningioma patients could be an interesting tool in a near future in a selected population to determine the tumor mutational change in recurrent high-grade meningiomas and to adapt the targeted therapy.

## Funding

The project leading to this publication has received funding from the Excellence Initiative of Aix Marseille University—A*Midex—a French “Investissement d’Avenir” program.

*Conflict of interest statement*. None declared.

## Authorship Statement

Conceptualization: A.B., C.R., P.R., and T.G..; methodology: A.B., C.R., P.R., N.B., and T.G.; experimentation: C.R., P.R., N.B., G.M., and A.B.; formal analysis: A.B., C.R., P.R., N.B.,G.M., and T.G.; investigation: S.F., M.M., K.F., S.B., C.P., E.T., and O.C.; resources and support: A.B., R.A., D.F.B., E.T., C.P., and O.C.; writing—original draft preparation: T.G., P.R., N.B., and A.B.; writing—review and editing: T.G. and A.B.; supervision: A.B., H.D., O.C., and D.F.B.; funding acquisition: A.B.

## References

[1] Bi WL, Greenwald NF, Abedalthagafi M, et al. Erratum: genomic landscape of high-grade meningiomas. NPJ Genom Med. 2017;2:26. 29263836 10.1038/s41525-017-0023-6 PMC5677977

[2] Weller M, Roth P, Sahm F, et al. Durable control of metastatic AKT1-mutant WHO grade 1 meningothelial meningioma by the AKT inhibitor, AZD5363. J Natl Cancer Inst. 2017;109(3):1–4. 10.1093/jnci/djw320 28376212

[3] Graillon T, Sanson M, Campello C, et al. Everolimus and octreotide for patients with recurrent meningioma: results from the phase II CEVOREM trial. Clin Cancer Res. 2020;26(3):552–557. 31969329 10.1158/1078-0432.CCR-19-2109

[4] Mansukhani S, Barber LJ, Kleftogiannis D, et al. Ultra-sensitive mutation detection and genome-wide DNA copy number reconstruction by error-corrected circulating tumor DNA sequencing. Clin Chem. 2018;64(11):1626–1635. 30150316 10.1373/clinchem.2018.289629 PMC6214522

[5] Xu C, Gu X, Padmanabhan R, et al. smCounter2: an accurate low-frequency variant caller for targeted sequencing data with unique molecular identifiers. Bioinformatics. 2019;35(8):1299–1309. 30192920 10.1093/bioinformatics/bty790 PMC6477992

[6] Piccioni DE, Achrol AS, Kiedrowski LA, et al. Analysis of cell-free circulating tumor DNA in 419 patients with glioblastoma and other primary brain tumors. CNS Oncol. 2019;8(2):CNS34. 30855176 10.2217/cns-2018-0015 PMC6713031

[7] Campos-Carrillo A, Weitzel JN, Sahoo P, et al. Circulating tumor DNA as an early cancer detection tool. Pharmacol Ther. 2020;207:107458. 31863816 10.1016/j.pharmthera.2019.107458 PMC6957244

[8] De Mattos-Arruda L, Mayor R, Ng CKY, et al. Cerebrospinal fluid-derived circulating tumour DNA better represents the genomic alterations of brain tumours than plasma. Nat Commun. 2015;6:8839. 26554728 10.1038/ncomms9839 PMC5426516

[9] Mahlokozera T, Vellimana AK, Li T, et al. Biological and therapeutic implications of multisector sequencing in newly diagnosed glioblastoma. Neuro Oncol. 2018;20(4):472–483. 29244145 10.1093/neuonc/nox232 PMC5909635

[10] McEwen AE, Leary SES, Lockwood CM Beyond the blood: CSF-derived cfDNA for diagnosis and characterization of CNS Tumors. Front Cell Dev Biol. 2020;8:45. 32133357 10.3389/fcell.2020.00045 PMC7039816

